# Endostatin in the pancreas

**DOI:** 10.1038/sj.bjc.6602180

**Published:** 2005-01-14

**Authors:** S J Wigmore

**Affiliations:** 1 Tissue Injury and Repair Group, MRC Centre for Inflammation Research, Medical School 6th Floor, University of Edinburgh, Teviot Place, Edinburgh EH8 9AG, UK

In this issue of the *British Journal of Cancer*, Brammer and co-workers investigate the status of endostatin expression in human pancreas cancer and normal pancreatic tissue. Endostatin was originally identified from the conditioned media of glioendothelioma cells as an inhibitor of angiogenesis (O'Reilly *et al*, 1997). These antiangiogenic properties immediately excited interest in the potential of endostatin as a modifier of cancer biology. Endostatin is derived as a proteolytic cleavage product from the C-terminus of the alpha chain of collagen XVIII, an extracellular matrix protein multiplexin (triple helix collagenous domain protein) encoded on human chromosome 21q22.3. Mutations of this region give rise to Knobloch syndrome, which is characterised by aberrant ocular development ascribed in part to a failure of regression blood vessels in the vitreous after birth and a failure of development of the retinal vasculature (Fukai *et al*, 2002).

In cancer biology, it has been demonstrated that endostatin can not only prevent the angiogenic switch early in tumour development but can also induce regression of some established tumours (Bergers *et al*, 1999). The same study also showed that the efficacy of endostatin in altering tumour vascularity could be potentiated by coadministration of other inhibitors of angiogenesis such as angiostatin. The mechanism of endostatin activity is still being elucidated; however, it seems likely that its effect may be mediated through more than one pathway (Abdollahi *et al*, 2004) and may also require the presence of other moieties such as e-selectin (Yu *et al*, 2004).

In a SCID mouse xenograft model, the human pancreatic carcinoma Hs-776T HS-W variant, which expresses endostatin, grows more slowly than the HS-R variant, which does not (Schuch *et al*, 2002). In the Hs-776 cell line, overexpression of vascular endothelial growth factor can overcome the inhibitory effect of endostatin on tumour growth, thus it seems likely that in this model, tumour growth is related to the balance between pro- and antiangiogenic factors. In another pancreas cancer xenograft model, rapidly growing L3.6pl tumours and slower growing BxPC3 were established in the peritoneal cavities of athymic nude mice, which were then treated with recombinant human endostatin (Raut *et al*, 2004). Endostatin resulted in reduced interleukin-8 expression in the animals and increased apoptosis rates in the L3.6pl tumours, but the actual tumour burden in terms of peritoneal carcinomatosis was increased compared with vehicle controls. Endostatin had less effect on the slower growing BxPC3 tumour. It is known that different xenograft models are regulated by different pathways, and that the necessary absence of a fully functional immune system in the host will also make these models somewhat artificial. It is clear however that endostatin can exert quite profound effects on xenografted human pancreas cancers and future work may improve our understanding of the scope and limitation of its use.

Brammer and co-workers found that endostatin was detectable in homogenates of resected human pancreatic cancers but not normal pancreas, and went on to demonstrate that in healthy pancreas endostatin is degraded by elastase but that the low or absent expression of elastase in pancreatic cancers is associated with stable endostatin expression. They hypothesise that the expression of endostatin in pancreatic tissues may in part explain the relatively avascular nature of these tumours. This relationship between the cancer and the inflammatory and stromal tissue surrounding it may therefore determine its biological behaviour. It seems counter-intuitive that a tumour should secrete an inhibitor of angiogenesis and the survival advantage of such a strategy is not immediately obvious. While we understand that an important part of the process of cancer invasion and metastasis requires angiogenesis, it is still possible that expression of an inhibitor of angiogenesis in an established tumour may be beneficial to the tumour. For example, tumours with poor vascularity may escape immunosurveillance to a greater degree than more vascular tumours and it is possible that the relatively poor response rates of pancreas cancers to conventional chemotherapy may also be associated with their avascular nature. Alternatively, chemotherapy may place a selection pressure on cancer cells selecting out clones, which by virtue of their inability to express elastase and break down endostatin, become relatively avascular. Such philosophy implies that a cancer attempts willingly or unwillingly to establish a survival benefit. This may be far too complex and the explanation may be much more straightforward. Expression of endostatin may simply arise as a consequence of the failure of pancreatic cancers to express the degradative enzyme elastase.

Since endostatin inhibits angiogenesis, it is interesting to speculate what the consequence would be if it were not present. Presumably, the tumour would have a more aggressive phenotype and it is not yet clear whether all pancreatic tumours do not express elastase or whether elastase expression may in some way determine pancreatic cancer behaviour through its effects on endostatin stability and expression. Perhaps, it is the balance between the expression of endostatin and elastase that is important in determining the phenotype of pancreas cancers. Whether surgical removal of a pancreas cancer also removes the inhibitory stimulus for metastases to grow is an interesting question. If this were the case and endostatin were to have any therapeutic benefit, this might be most evident in an adjuvant surgical model. In this context, the benefits of an angiogenesis inhibitor in preventing tumour metastasis would have to be balanced with the risks of potentially delaying wound healing.

The relative instability of endostatin may limit its usefulness as a cancer treatment; however, it has been documented that continuous infusion can improve its efficacy over bolus treatment (Kisker *et al*, 2001). Now that at least one inhibitor of endostatin is known, it may also be possible to modify the amino-acid sequence of endostatin to create analogues, which retain antiangiogenic activity but which are more resistant to degradation by pancreatic elastase. One issue which was not addressed by the study by Brammer *et al* (2005) is whether any products of endostatin resulting from elastase digestion retain activity as inhibitors of angiogenesis. Adenocarcinoma of the pancreas has largely frustrated the efforts of oncologists to modify its behaviour. The failure of pancreatic cancers to elaborate elastase and the consequent inability to degrade the angiogenesis inhibitor endostatin may represent a chink in the armour of pancreas cancer upon which an effective treatment regimen can be designed.
